# Silencing Aurora-kinase-A (AURKA) reinforced the sensitivity of diffuse large B-cell lymphoma cells to cyclophosphamide, doxorubicin, vincristine, and prednisone (CHOP) via suppressing β-Catenin and RAS-extracellular signal-regulated protein kinase (ERK1/2) pathway

**DOI:** 10.1080/21655979.2021.1985346

**Published:** 2021-10-17

**Authors:** Shaoxiong Wang, Li Sun

**Affiliations:** Department of Hematology, Quanzhou First Hospital, Quanzhou City, Fujian Province, China

**Keywords:** Diffuse large B-cell lymphoma (DLBCL), cyclophosphamide, doxorubicin, vincristine, and prednisone (CHOP), Aurora-kinase-A (AURKA), apoptosis

## Abstract

The therapeutic effects of standard cyclophosphamide, doxorubicin, vincristine and prednisone (CHOP) therapy for prevalent lymphoma diffuse large B-cell lymphoma (DLBC, DLBCL) still require improvement. Cancer-related aurora-kinase-A (AURKA) may work as a target for DLBCL treatment and its effect on CHOP therapy was investigated in the present study. The Gene Expression Profiling Interactive Analysis 2 was applied to analyze AURKA expression in DLBC tumor tissues and normal lymphoid tissues. The DLBCL tissues and normal lymphoid tissues were obtained from the DLBCL patients and healthy volunteers. Clinic data of patients were recorded, and AURKA expression in tissues and cells was detected and analyzed using quantitative real-time polymerase chain reaction (qRT-PCR) and immunohistochemistry. After AURKA in DLBCL cells was silenced or overexpressed and treated with CHOP, viability and apoptosis were detected by Cell Counting Kit-8 (CCK-8) assay and flow cytometry. Expressions of AURKA, β-Catenin, phosphorylated (p)-β-Catenin, extracellular signal-regulated protein kinase (ERK1/2), p-ERK1/2 and RAS were detected using qRT-PCR and Western blot. AURKA was highly expressed in DLBCL tissues and cells. Silencing AURKA inhibited AURKA expression and viability, but promoted apoptosis of DLBCL cells. CHOP had no obvious effects on AURKA expression while reducing viability and promoting apoptosis of DLBCL cells. Silencing AURKA enhanced the effects of CHOP on cell apoptosis of DLBCL cells by inhibiting the expressions of RAS and β-Catenin as well as the ratio of p-ERK1/2/ERK1/2 and promoting the ratio of p-β-Catenin/β-Catenin. Silencing AURKA reinforced the therapeutic effects of CHOP on reducing viability and promoting apoptosis of DLBCL cell via repressing β-Catenin and RAS-ERK1/2 pathway.

## Introduction

Lymphomas are lymphocytic tumors arising from the lymphatic system and can be evolved from mutations in B lymphocytes of different stages [1]. Dynamic genetic alteration will occur in the process of activation and development of B-cells and the translocation of chromosome and mutations of genome in genetic alteration would result in the tumorigenesis of B lymphoma [2]. In the United State in 2020, there were 85 thousand new cases diagnosed with lymphoma and 20 thousand cases of death [3]. Diffuse large B-cell lymphoma (DLBC, DLBCL) is the most prevalent type of non-Hodgkin lymphoma, and patients with this disease account for one-third of the lymphoma cases [4]. The standard method for DLBCL treatment is the immunochemotherapy, but its prognosis is still poor [5–7]. Therefore, a novel immunochemotherapy with better efficacy and security is necessary for DLBCL treatment.

Immunochemotherapy is a standard therapeutic method for DLBCL treatment and the therapy of cyclophosphamide, doxorubicin, vincristine, and prednisone (CHOP) is invented for non-Hodgkin lymphoma-like DLBCL [8,9]. According to a previous study, CHOP could improve the prognosis for the DLBCL patients [10], but the specific treatment of CHOP therapy for kids is necessary because the prognosis of CHOP on kids is usually poor when compared with that of adults [11]. CHOP treatment could cause great gonadal damage. In addition to that, the resistance of DLBCL to CHOP therapy is still a great issue [12,13]. Targets to improve the therapeutic effects of CHOP therapy on DLBCL are urgently needed.

As a member of serine/threonine kinase family, Aurora-kinase-A (AURKA) participates in biological processes about mitosis and other non-mitosis activities [14]. Overexpressed AURKA is discovered in various cancers, such as in colorectal, breast, gastric, ovarian, esophageal and liver cancers [15]. According to a recent study, some oncogenic factors such as β-Catenin could be mediated and activated by the overexpression of AURKA [15]. The pathway of extracellular signal-regulated protein kinase (ERK1/2) in cancers, such as breast cancer, squamous cell carcinoma of head and neck cancer, could be activated by AURKA as well, thereby promoting the motility of cancer cells as well as proliferation and growth of cancer [16,17]. Furthermore, a previous study also demonstrated that the AURKA has the ability to enhance the Wnt and RAS signaling, which are usually hyperactivated in the colorectal cancer [18]. In DLBCL, inhibition of Aurora kinase could promote the apoptosis of DLBCL cells [19]. The alisertib, a kind of AURKA inhibitor, also presents a preclinical synergy with rituximab and vincristine for the treatment of DLBCL [20]. However, the effects of AURKA on the CHOP therapy for DLBCL have not been fully elucidated.

Therefore, we hypothesized that the inhibition of AURKA may be beneficial to the treatment of DLBCL by CHOP and verified whether silencing AURKA can enhance the therapeutic effect of CHOP therapy on DLBCL by regulating β-catenin and RAS-ERK1/2 pathway *in vitro*.

## Material and methods

### Ethic statement

Ethics committee of Quanzhou First Hospital (approval number: XYNK20200513) had reviewed and approved this research. Patients had signed the written informed consents and agreed to use their tissues.

### Gene expression profiling interactive analysis 2 (GEPIA2) analysis

The expression of AURKA in DLBC tissues and normal tissues was analyzed by the GEPIA2 (Both TCGA and GTEX data were used to analyze AURKA) [21] (http://gepia2.cancer-pku.cn/#index).

### Tissue samples

The samples of DLBCL tissues were obtained from 60 DLBCL patients, and the normal lymphoid tissues for control were isolated from the 26 healthy volunteers from 2019 August to 2020 May in Quanzhou First Hospital. All of the patients and volunteers had not received any radiotherapy or chemotherapy treatment. There were no other infectious diseases, cancers, autoimmune diseases or so on in the patients. After primary DLBCL cancer tissues and normal tissues were isolated by operation, they were immediately frozen by liquid nitrogen and then preserved at −80°C as previously described [22].

### Clinic data analysis

The DLBCL tissues were isolated from 60 DLBCL patients. After the detection of the difference of AURKA expression among DLBCL tissues by quantitative real-time polymerase chain reaction (qRT-PCR), the obtained median of AURKA expression levels in 60 patients was considered as the boundary in distinguishing the low AURKA expression and high AURKA expression. Hence, there were 30 patients with low AURKA expression and 30 patients with high AURKA expression. According to the reference of previous study [23], the relationship between AURKA expression and the clinical features of patients, including gender, age, cell of origin, plasmablastic subtype, largest tumor size, clinical stages, lactate dehydrogenase (LDH) level and number of extranodal sites, were explored in our study and presented in Table 1.

**Table 1.** The relationship between AURKA expression and clinical characteristics

Variable	n	AURKA expression		P value
		Low	High	
Total	60	30	30	
Gender				
Male	35	18	17	0.793
Female	25	12	13	
Age				
≤60	26	15	11	0.297
>60	34	15	19	
Cell of origin				
GCB	36	21	15	0.114
non-GCB	24	9	15	
Plasmablastic subtype				
No	33	17	16	0.795
Yes	27	13	14	
Largest tumor size (cm)				
≤5	33	22	11	0.004
>5	27	8	19	
Stage				
I–II	34	21	13	0.037
III–IV	26	9	17	
LDH level				
Normal	27	12	15	0.436
Elevated	33	18	15	
No. of extranodal sites				
<2	25	14	11	0.432
≥2	35	16	19	

AURKA: Aurora kinase A; GCB: germinal center B-cell; LDH: lactate dehydrogenase.

### Immunohistochemistry (IHC)

The AURKA level was also detected by the IHC assay [24,25]. In detail, the lymphoid tissues were fixed by 4% Paraformaldehyde (P0099-100 ml; Beyotime, Shanghai, China) for 10 minutes (min) and made into paraffin slices of 3 μm by dehydration with graded ethanol (70%, 80%, 90%; E7023; Sigma-Aldrich, St. Louis, MO, USA) and embeddedness into paraffin (1.07174; Sigma-Aldrich, St. Louis, MO, USA). Xylene I and II (214,736; Sigma-Aldrich, St. Louis, MO, USA) and graded ethanol (100%, 95%, 90%, 80%, 70%) were separately applied to the dewaxed paraffin slices for 5 min and the rehydrated slices for 3 min. After 20 min of treatment with 3% hydrogen peroxide (88,597; Sigma-Aldrich, St. Louis, MO, USA), the edetate disodium (EDTA) buffer (E1161; Sigma-Aldrich, St. Louis, MO, USA) was used to pretreat the tissues in a pressure cooker at 100°C for 2  min, and then the tissues were incubated with 10% goat serum (ab7481; Abcam, Cambridge, UK) for 10  min at room temperature. Next, the tissues were incubated with the anti-AURKA antibody (1:1000; ab61114; Abcam, Cambridge, UK) at 4°C for 12 hours (h). After incubation with goat anti-rabbit IgG H&L secondary antibody (1:1000; ab205718; Abcam, Cambridge, UK) at room temperature for 1 h, the tissues were treated with chromogenic reagent Diaminobenzidine (P0203; Beyotime, Shanghai, China). Subsequently, the hematoxylin (H9627; Sigma-Aldrich, St. Louis, MO, USA) was used to counterstain the tissues. After further dehydration and mounting, the results were observed under a fluorescence microscope (Zeiss Axio Scope A1; Zeiss, Oberkochen, Germany) under the ×100 magnification.

### Cell culture and CHOP treatment

The normal B lymphocyte GM12878 (GM12878; Coriell, Camden, NJ, USA) was cultured in Roswell Park Memorial Institute 1640 (RPMI-1640) Medium (A4192301; Gibco, Carlsbad, CA, USA) supplemented with 15% fetal bovine serum (FBS; C0227; Beyotime, Shanghai, China) and 2 mM L-glutamine (G8540; Sigma-Aldrich; St. Louis, MO, USA) [26]. DLBCL cell lines (OCI-LY18 (ACC 699), OCI-LY19 (ACC 528), OCI-LY1 (ACC 722), OCI-LY3 (ACC 761) and OCI-LY7 (ACC 688)), which were purchased from Deutsche Sammlung von Mikroorganismen und Zellkulturen (DSMZ; Braunschweig, Germany) and Farage (CRL-2630; American Type Culture Collection (ATCC), Manassas, VA, USA), were cultured in the RPMI-1640 Medium supplemented with 10% FBS and 1% Penicillin-Streptomycin Solution (C0222; Beyotime, Shanghai, China) and 2 mM L-glutamine (G8540; Sigma-Aldrich, St. Louis, MO, USA) [27]. The incubation condition was set at 37°C with 5% CO_2_.

The CHOP treatment *in vitro* was carried out following the previous reference [13]. As OCI-LY7 and Farage showed a higher expression of AURKA, the OCI-LY7 and Farage cells in 96-well plates were cultured at 37°C for 24 h and then treated with CHOP at the concentrations of 0, 10, 20 and 40 ng/mL, respectively. The ratio of the four drugs purchased from Sigma-Aldrich (St. Louis, MO, USA) in CHOP was as follows: 80 mg of cyclophosphamide (PHR1404)/5.5 mg of doxorubicin (D1515)/0.16 mg of vincristine (V0405000)/11.1 mg of prednisone (P-122).

### Transfection

For transfection [28], the small interfering RNA targeting AURKA (siAURKA; sense obligo: 5ʹ-GGUGUCUAGUUAUUAACAAAC-3; anti-sense obligo: 5ʹ-UUGUUAAUAACUAGACACCUG-3) or siAURKA negative control (siNC; sense obligo: 5ʹ-UGGCUAGUGUAUCAUAUAAAC-3; anti-sense obligo: 5ʹ-CUUAAGUGACAUUACCUGUAA-3) used in the present study was synthesized by the GenePharma (Shanghai, China). Concretely, OCI-LY7 and Farage cells at a density of 1 × 10^5^ cells/well were separately seeded in 6-well plates to 80% confluence. By using Lipofectamine 2000 transfection reagent (11,668,019; Thermo Fisher, Waltham, MA, USA), the DLBCL cells were transfected with siAURKA or siNC at 37°C. 48 h after transfection, the cells were harvested.

### Cell counting kit-8 (CCK-8) assay

In the present study, the CCK-8 assay was used to detect cell viability [29]. The 96-well plates were plated with OCI-LY7 and Farage cells at a density of 5 × 10^3^ cells/well. After being cultured for 24 h and 48 h, the cells were separately added with 10 μL of CCK-8 kit (ab228554; Abcam, Cambridge, UK) and then were incubated in the dark for 4 h. Finally, the absorbance was measured and recorded at a wavelength of 460 nm using a Molecular Devices SpectraMax®i3 microplate reader (Molecular Devices, San Jose, CA, USA).

### RNA isolation and qRT-PCR

Total RNA Isolation Kit (A27828; Thermo Fisher, Waltham, MA, USA) was applied to extract the RNAs from tissues and cells, and these RNAs were preserved at −80°C. The Nano Drop 2000 biological spectrometer (ND-2000; Thermo Fisher, Waltham, MA, USA) was used to measure the concentration of RNAs. By using Maxima H Minus First Strand cDNA Synthesis Kit (K1651; Thermo Fisher, Waltham, MA, USA), complementary DNAs (cDNAs) were synthesized from 1 μg of RNA. QRT-PCR assay was carried out with Fast SYBR™ Green Master Mix (4,385,610; Thermo Fisher, Waltham, MA, USA) in 7500 Fast Real-Time PCR System (4,351,106; Thermo Fisher, Waltham, MA, USA). The primers are listed in Table 2.

**Table 2.** Primer for qRT-PCR

	Forward Primer(5ʹ->-3ʹ)	Reverse Primer(5ʹ->-3ʹ)
AURKA GATATCTCAGTGGCGGACG GAPDH AGGTCGGTGTGAACGGATTTG		GCAATGGAGTGAGACCCTCT GGGGTCGTTGATGGCAACA

The conditions of qRT-PCR were listed as follows: activating at 95°C for 15 min, followed by 40 cycles of denaturizing at 95°C for 15 second (s) and a combined annealing/extension at 60°C for 1 min [30]. GAPDH was used as the internal control in the present study. Besides, 2^−ΔΔCT^ calculation method was applied to quantify the expression of relative genes [31].

### Flow cytometry

The apoptosis of OCI-LY7 and Farage cells was determined by flow cytometry using Annexin V-fluorescein Isothiocyanate (FITC)/propidium iodide (PI) cell apoptosis kit (C1062S; Beyotime, Shanghai, China) [32]. All the operations were performed under the guidance of manufacturer’s instructions. After being transfected with siNC or siAURKA, treated with CHOP and centrifuged at 300 × *g* for 5 min, 1 × 10^5^ DLBCL cells were resuspended in 100 μL Binding Buffer of the kit, and 1 × 10^6^ cells/mL cell solution was obtained. Next, 5 μL Annexin V-FITC and PI working solution was used to incubate the cells at room temperature for 15 min without light. The apoptosis of cells was measured using the CytoFLEX Flow Cytometer (C02945; Beckman Coulter, Indianapolis, IN, USA), and the data were analyzed using Kaluza Analysis Software version 3.1 (Beckman Coulter, Indianapolis, IN, USA).

### Western blot

The relative protein expression levels of AURKA, β-Catenin, phosphorylated (p)-β-Catenin, ERK1/2, p-ERK1/2, RAS in the OCI-LY7 and Farage cells were quantified by Western blot [33,34]. The harvested cells were lysed with Radio Immunoprecipitation Assay (RIPA) lysis buffer (89,901; Thermo Fisher, Waltham, MA, USA) and centrifuged at 12,000 × *g* at 4°C for 20 min. After the relative protein concentrations of supernatant were measured by Pierce™ Bicinchoninic acid (BCA) Protein assay kit (23,225; Thermo Fisher, Waltham, MA, USA), the protein in obtained supernatant was degenerated and preserved at −20°C. After being electrophoresed by sodium dodecyl sulfate-polyacrylamide gel electrophoresis (SDS-PAGE) constructed by the TruPAGE™ Precast Gels (PCG2004; Sigma-Aldrich, St. Louis, MO, USA) and TruPAGE™ TEA-Tricine SDS Running Buffer (PCG3001; Sigma-Aldrich, St. Louis, MO, USA), 50 μg of protein was transferred into Polyvinylidene fluoride membrane (PVDF; FFP33; Beyotime, Shanghai, China). The membranes were blocked with 5% skimmed milk at room temperature for 2 h and incubated with the primary antibodies at 4°C overnight, mainly including those antibodies against β-Catenin (ab32572; 92 kDa; 1:1000), p-β-Catenin (ab27798; 92 kDa; 1:1000), RAS (ab52939; 21 kDa; 1:1000), ERK1/2 (ab50011; 42–44 kDa; 1:1000), p-ERK1/2 (ab17942; 42–44 kDa; 1:1000) and GAPDH (ab181602, 36 kDa; 1:1000) purchased from Abcam in Cambridge, UK. GAPDH was used as the internal control. After 24-h incubation, the membranes were incubated with goat anti-rabbit secondary antibody (ab205718; 1:2000; Abcam, Cambridge, UK) at room temperature for 1 h, and then rinsed with Tris-buffered saline Tween (TBST; 91,414; Sigma-Aldrich, St. Louis, MO, USA) for 3 times. Subsequently, the membranes were incubated with an enhanced chemiluminescence (ECL) kit (WP2005; Thermo Fisher, Waltham, MA, USA) for visualization. After the membranes were exposed in iBright™ CL1500 Imaging System (A44240; Invitrogen, Carlsbad, CA, USA), the gray value of the protein bands was quantified by Image J (version 5.0; Bio-Rad, Hercules, CA, USA).

### Statistical analysis

All the experiments of this study were conducted 3 times repeatedly. The experimental data were displayed as Mean ± standard deviation (SD). Statistical analysis was performed in SPSS 22.0 (IBM Cor., Armonk, NY, USA). One-way analysis of Variance (ANOVA) was used, followed by the Bonferroni adjustment to determine statistical significance (*P* < 0.05).

## Results

The current research hypothesized that inhibition of AURKA may be beneficial to the treatment of DLBCL by CHOP, and our objective was to confirm whether siAURKA enhanced the sensitivity of DLBCL to CHOP therapy and the molecular mechanism of AURKA. The current findings revealed that AURKA expression was upregulated in DLBCL tissues and cells, and siAURKA strengthened the effects of CHOP on enhancing the apoptosis of DLBCL cells. Functionally, siAURKA reinforced the effect of CHOP via β-Catenin and RAS-ERK1/2 pathway.

### AURKA showed a high expression in DLBCL tissues and cells

**Figure 1.**
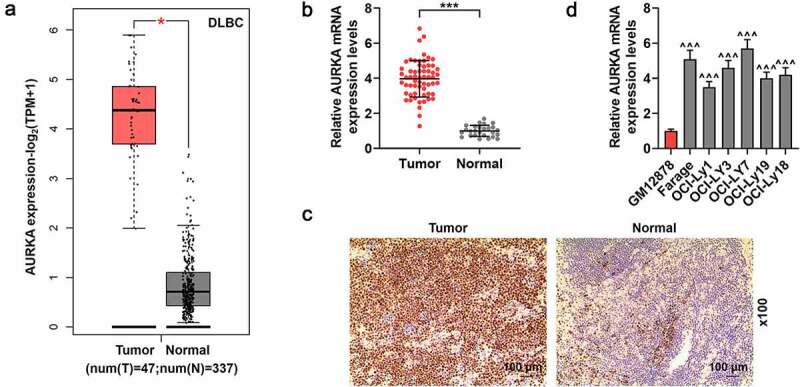
AURKA presented a high expression in DLBCL tissues and cells. (a) The expression of AURKA in the DLBC tumor tissues from DLBC patients than that in normal lymphoid tissues from healthy people was analyzed by GEPIA2 (http://gepia2.cancer-pku.cn/#index). (b) The expression of AURKA in DLBCL tumor tissues from DLBCL patients and normal lymphoid tissues from healthy volunteers was detected using qRT-PCR, and the GAPDH was the internal control. (c) The expression of AURKA in DLBCL tumor tissues from DLBCL patients and normal lymphoid tissues from healthy volunteers was detected using immunohistochemistry (IHC) assay (magnification, ×100). (d) The expression of AURKA in DLBCL cells and normal B lymphocyte was detected using qRT-PCR, and the GAPDH was the internal control. All experiments were repeatedly performed over 3 times. Experimental data were expressed by mean ± standard deviation (SD). (*P < 0.05, ***P < 0.001; ^^^^^*P* < 0.001; * vs. normal group; ^^^ vs. GM12878 group) (AURKA: Aurora-kinase-A; DLBCL: diffuse large B-cell lymphoma; GEPIA2:gene expression profiling interactive analysis 2; qRT-PCR: quantitative real-time polymerase chain reaction)

The expression of AURKA in the DLBCL was determined in the present study. According to the analysis from GEPIA2 in Figure 1(a), the AURKA presented a higher expression in DLBC tumor tissues from DLBC patients than that in normal lymphoid tissues from healthy people (Figure 1(a); *P* < 0.05). The expression of AURKA in DLBCL tumor tissues from DLBCL patients and normal lymphoid tissues from healthy volunteers was detected using qRT-PCR and IHC assays. Figures 1(b,c) showed that AURKA was highly expressed in DLBCL tumor tissues and compared with those in normal tissues, there were more AURKA-positive cells in DLBCL tumor tissues (Figures 1(b,c); *P* < 0.001).

Furthermore, the expression of AURKA in DLBCL cells and normal lymphocyte was also quantified using qRT-PCR. As shown in Figure 1(d), compared with that in normal B lymphocyte GM12878, AURKA expression was higher in the DLBCL cells (Figure 1(d); *P* < 0.001), suggesting that AURKA presented a high expression in DLBCL tissues and cells.

According to the clinic data of DLBCL patients with different AURKA expressions in Table 1, the high AURKA expression was involved in the tumor size and stage of DLBCL (*P* < 0.05).

### Silencing AURKA downregulated AURKA expression, reduced cell viability of DLBCL cells and promoted the DLBCL cells apoptosis

**Figure 2.**
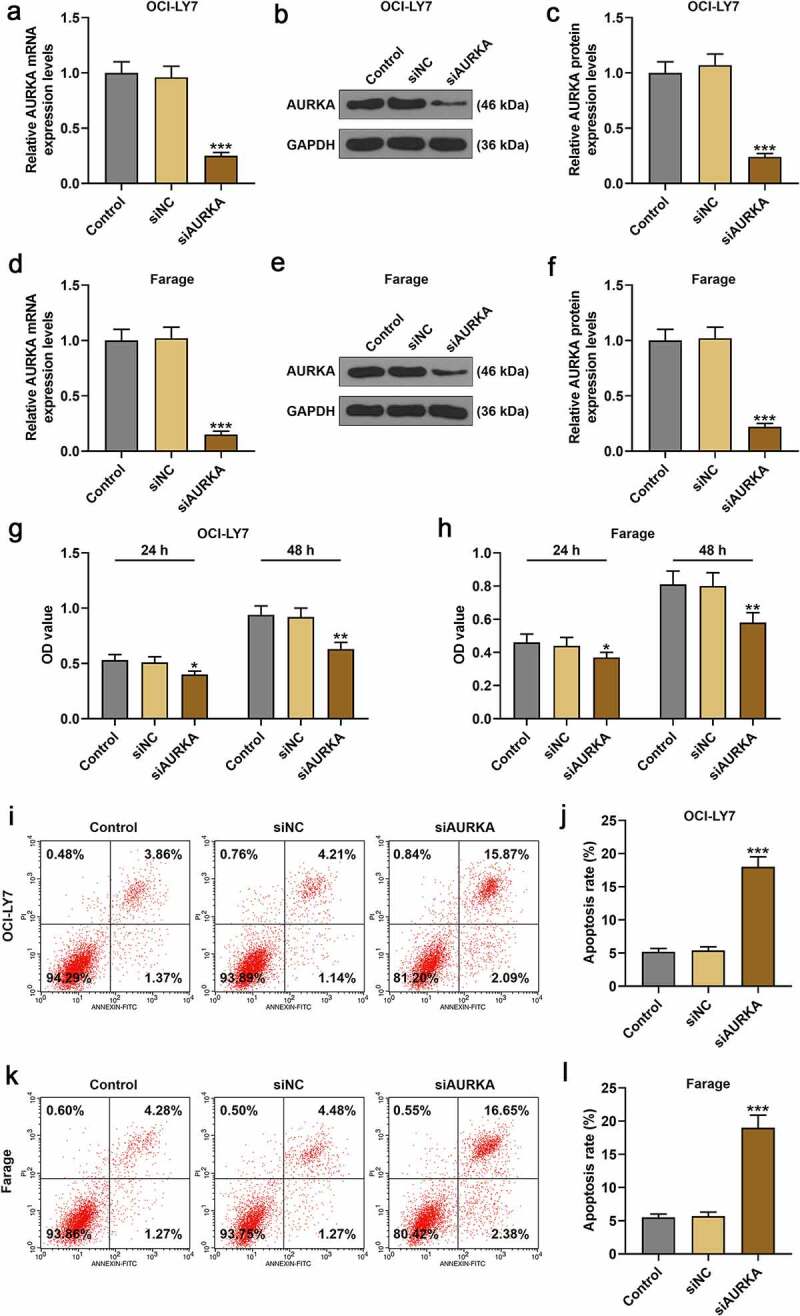
Silencing AURKA negatively regulated AURKA expression and viability of DLBCL cells, and promoted the apoptosis of DLBCL cells. (a) The mRNA expression of AURKA in OCI-LY7 cells transfected with siAURKA or siNC was detected by qRT-PCR, and the GAPDH was internal control. (b-c) The protein expression of AURKA in OCI-LY7 cells transfected with siAURKA or siNC was detected by Western blot, with GAPDH as the internal control. (d) The mRNA expression of AURKA in Farage cells transfected with siAURKA or siNC was detected by qRT-PCR, and the GAPDH was used as the internal control. (e-f) The protein expression of AURKA in Farage cells transfected with siAURKA or siNC was detected by Western blot, and the GAPDH was the internal control. (g-h) The cell viability of OCI-LY7 (g) and Farage (h) cells transfected with siAURKA or siNC and cultured for 24 h and 48 h was detected by CCK-8 assay. (i-l) The cell apoptosis of OCI-LY7 (i-j) and Farage (k-l) cells transfected with siAURKA or siNC was detected by flow cytometry. All experiments were repeatedly performed over 3 times. Experimental data were expressed by mean ± standard deviation (SD). (*P < 0.05, **P < 0.01, ***P < 0.001; * vs. siNC group) (AURKA: Aurora-kinase-A; DLBCL: diffuse large B-cell lymphoma; siAURKA: short interfering RNA targeting AURKA; siNC: negative control of siAURKA; qRT-PCR: quantitative real-time polymerase chain reaction; CCK-8: cell counting kit 8; h: hours)

Since AURKA was detected to present a higher expression in Farage and OCI-LY7 cells among DLBCL cells in the experiments above, the Farage and OCI-LY7 cells were transfected with siAURKA or siNC to unveil the effects of AURKA on DLBCL cells in the following study. As shown in Figure 2(a,d), compared with DLBCL cells transfected with siNC, the AURKA expression was downregulated in DLBCL cells transfected with siAURKA (Figure 2(a,d); *P* < 0.001), revealing that siAURKA was successfully transfected.

After transfection with siAURKA or siNC, the cell viability and apoptosis of DLBCL cells were detected. As profiled in Figure 2(g,h), the viability of DLBCL cell transfected with siAURKA and cultured for 24 h and 48 h was reduced compared with the DLBCL cells transfected with siNC (Figure 2(g,h); *P* < 0.05). As for the apoptosis of DLBCL cells, the apoptosis of DLBCL cells in siAURKA group was promoted compared with that of the cells in siNC group (Figures 2(i,l); *P* < 0.001), manifesting that silencing AURKA negatively regulated AURKA expression as well as cell viability of DLBCL cells, and promoted the DLBCL cell apoptosis.

### CHOP repressed the cell viability and had no obvious effects on AURKA expression in DLBCL cells

**Figure 3.**
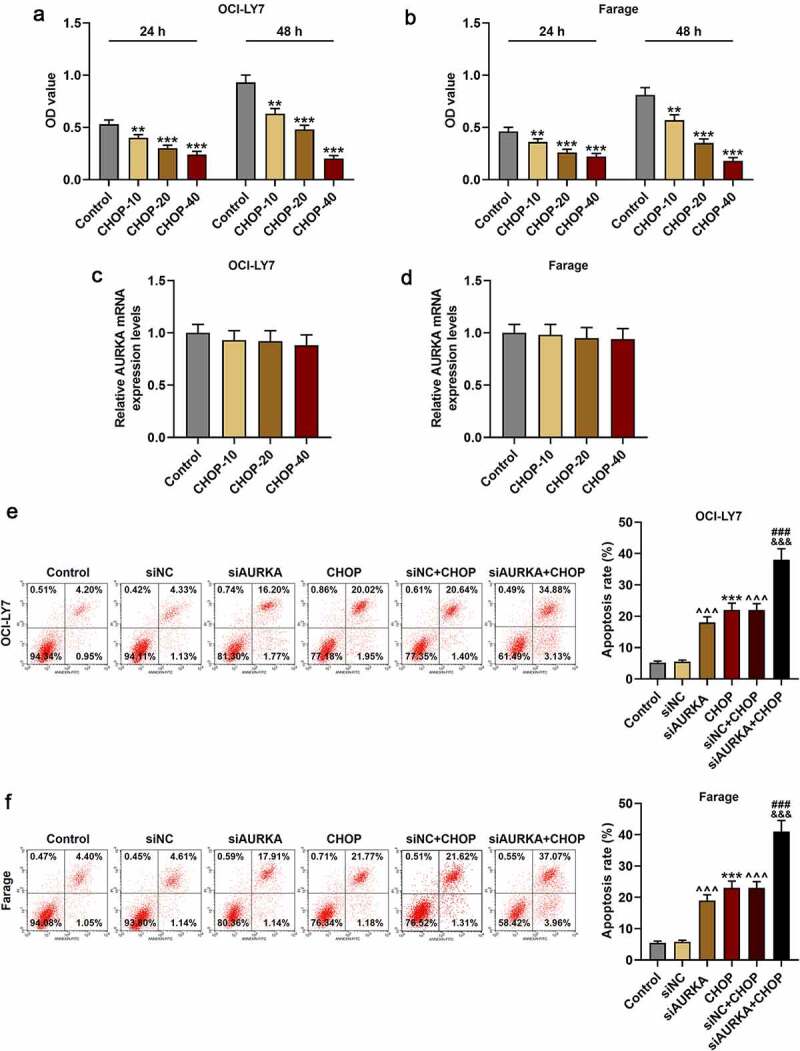
CHOP reduced the viability and promoted apoptosis of DLBCL cells, and silencing AURKA enhanced the effects of CHOP on DLBCL cell apoptosis. (a-b) After treatment with CHOP (0, 10, 20 and 40 ng/mL) and cultured for 24 h and 48 h, the cell viability of OCI-LY7 (a) and Farage (b) cells was detected by CCK-8 assay. (c-d) After treatment with CHOP (0, 10, 20 and 40 ng/mL), the expression of AURKA in OCI-LY7 (c) and Farage (d) cells was detected by qRT-PCR, and GAPDH worked as internal control. (e-f) After transfection with siAURKA or siNC and treatment with CHOP (20 ng/mL), the apoptosis of OCI-LY7 (e) and Farage (f) cells was detected by flow cytometry. All experiments were repeatedly performed over 3 times. Experimental data were expressed by mean ± standard deviation (SD). (**P < 0.01, ***P < 0.001; ^^^^^*P* < 0.001; ^###^*P* < 0.001; ^&&&^*P* < 0.001; * vs. Control group; ^^^ vs. siNC group; ^#^ vs. siAURKA group; ^&^ vs. siNC+CHOP group) (CHOP: cyclophosphamide, doxorubicin, vincristine, and prednisone; AURKA: Aurora-kinase-A; DLBCL: diffuse large B-cell lymphoma; qRT-PCR: quantitative real-time polymerase chain reaction; siAURKA: short interfering RNA targeting AURKA; siNC: negative control of siAURKA; CCK-8: cell counting kit 8; h: hours)

After the DLBCL cells were treated with CHOP (0, 10, 20 and 40 ng/mL) and cultured for 24 h and 48 h, the cell viability was detected again. In Figure 3(a,b), it could be found that compared with that of Control group, the cell viability was suppressed in CHOP group (Figure 3(a,b); *P* < 0.001).

Moreover, the AURKA expression of the cells treated with CHOP (0, 10, 20 and 40 ng/mL) was also detected, and there was no obvious difference in AURKA expression between cells in Control group and CHOP group (Figure 3(c,d)), denoting that CHOP suppressed the cell viability and had no obvious effects on AURKA expression in DLBCL cells.

### Silencing AURKA strengthened effects of CHOP on enhancing DLBCL cells apoptosis

Because the inhibitory effects of 40 ng/mL CHOP on DLBCL cells were strong and CHOP of 20 ng/mL had obvious effects on reducing viability of DLBCL cells, the CHOP of 20 ng/mL was used in the following experiments. The apoptosis of DLBCL cells was detected following transfection with siNC or siAURKA and treatment with CHOP of 20 ng/mL. As displayed in Figure 3(e,f), the apoptosis of DLBCL cell was increased in CHOP group as compared with that in Control group (Figure 3(e,f); *P* < 0.001), and the apoptosis of DLBCL cells in siAURKA group and siNC+CHOP group was enhanced in comparison with that in siNC group (Figure 3(e,f); *P* < 0.001). Meanwhile, the apoptosis of DLBCL cell in siAURKA+CHOP group was increased when compared with those in siAURKA group and siNC+CHOP group (Figure 3(e,f); *P* < 0.001), signifying that CHOP enhanced the apoptosis of DLBCL cells and silencing AURKA strengthened effects of CHOP on enhancing the apoptosis of DLBCL cells.

### Silencing AURKA downregulated the AURKA and RAS expressions, reduced ratio of p-ERK1/2/ERK1/2 yet upregulated the ratio of p-β-Catenin/β-Catenin, and these effects were reinforced by CHOP

**Figure 4.**
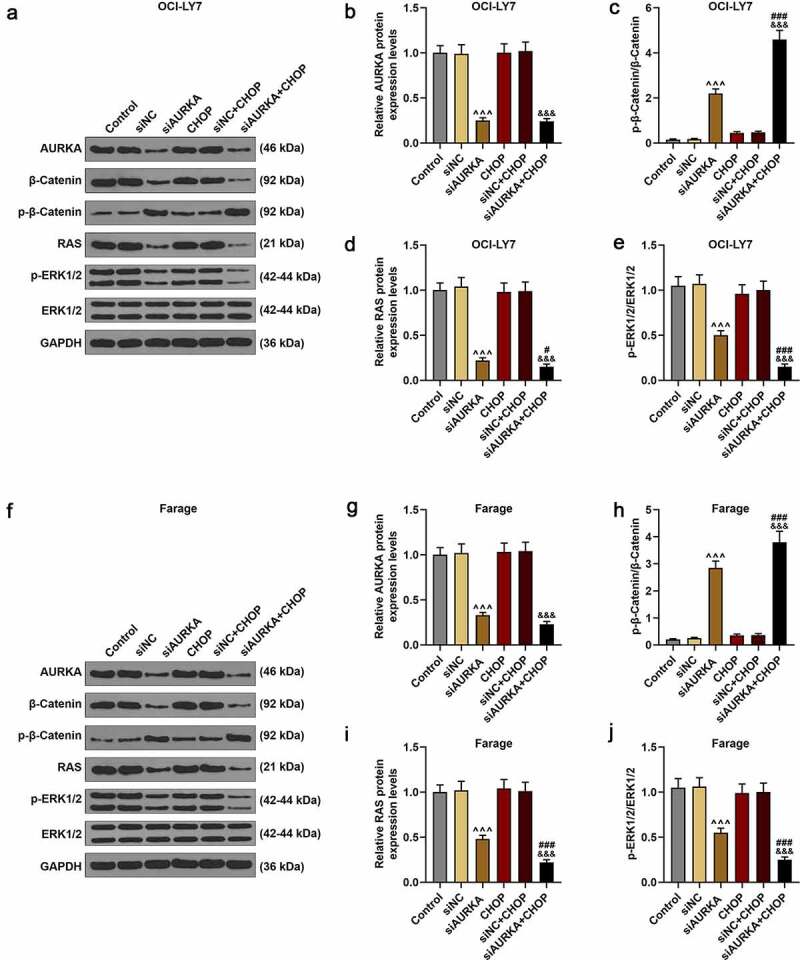
Silencing AURKA downregulated the AURKA and RAS expressions, reduced the ratio of p-ERK1/2/ERK1/2 yet increased the ratio of p-β-Catenin/β-Catenin, and these effects were reinforced by CHOP. (a-j) After the OCI-LY7 (a-e) and Farage (f-j) cells were transfected with siAURKA or siNC and treated with CHOP (20 ng/mL), the protein expressions of AUKA, RAS, p-β-Catenin, β-Catenin, p-ERK1/2 and ERK1/2 were detected using Western blot and the ratios of p-β-Catenin/β-Catenin and p-ERK1/2/ERK1/2 were analyzed, with GAPDH used as an internal control. All experiments were repeatedly performed over 3 times. Experimental data were expressed by mean ± standard deviation (SD). (^^^^^*P* < 0.001; ^#^*P* < 0.05, ^###^*P* < 0.001; ^&&&^*P* < 0.001; ^^^ vs. siNC group; ^#^ vs. siAURKA group; ^&^ vs. siNC+CHOP group) (CHOP: cyclophosphamide, doxorubicin, vincristine, and prednisone; AURKA: Aurora-kinase-A; DLBCL: diffuse large B-cell lymphoma; siAURKA: short interfering RNA targeting AURKA; siNC: negative control of siAURKA; ERK: extracellular-signal regulated kinase; p-ERK: phosphorylated-ERK)

The expressions of AURKA, p-β-Catenin, β-Catenin, RAS, p-ERK1/2 and ERK1/2 were detected using qRT-PCR and the ratios of p-β-Catenin/β-Catenin and p-ERK1/2/ERK1/2 were calculated (Figure 4(a,j)). In Figure 4(a,b,d,f,g,i), it could be found that the expressions of AURKA and RAS were decreased in siAURKA group relative to that in Control group (Figure 4(a,b,d,f,g,i); *P* < 0.001); and in siAURKA+CHOP group, the AURKA expression was declined as compared with that in siNC+CHOP group and the RAS expression was diminished when compared with those in siAURKA group and siNC+CHOP group (Figure 4(a,b,d,f,g,i); *P* < 0.05).

As for the ratios of p-β-Catenin/β-Catenin and p-ERK1/2/ERK1/2, compared with Control group, the ratio of p-β-Catenin/β-Catenin was increased in siAURKA group (Figure 4(a,c,f,h); *P* < 0.001). In siAURKA+CHOP group, the ratio of p-β-Catenin/β-Catenin was elevated compared with those in siAURKA group and siNC+CHOP group (Figure 4(a,c,f,h); *P* < 0.001). Compared with that in Control group, the ratio of p-ERK1/2/ERK1/2 was reduced in siAURKA group (Figure 4(a,e,f,j); *P* < 0.001). In siAURKA+CHOP group, the ratio of p-ERK1/2/ERK1/2 was reduced compared with those in siAURKA group and siNC+CHOP group (Figure 4(a,e,f,j); *P* < 0.001). In CHOP group and siNC+CHOP group, there were no obvious changes in the expressions of AURKA and RAS as well as the ratios of p-β-Catenin/β-Catenin and p-ERK1/2/ERK1/2, implicating that silencing AURKA reinforced the therapeutic effects of CHOP on DLBCL via suppressing the β-Catenin pathway and RAS-ERK1/2 pathway.

## Discussion

DLBCL, which is the most prevalent subtype of the non-Hodgkin lymphoma in the world, accounts for about one-third of patients diagnosed as lymphoma [35]. The standard method for DLBCL treatment is the immunochemotherapy, which improves the overall survival rate of patients to 60% [6]. Although the immunochemotherapy of DLBCL has the potential to be applied in the patients more than 80 years old, the required conditions are strict [36]. For a subset of DLBCL patients, the prognosis of immunochemotherapy is still poor and the resistance to immunochemotherapy remains a great challenge [5–7]. Thus, the target for improving the efficacy of immunochemotherapy on DLBCL is necessary.

The functions of AURKA in regulating mitosis, spindle composition and centrosome have been well researched [37]. In addition, studies have reported that AURKA from the serine/threonine kinase family was overexpressed in various cancers and can be used as a prognostic marker for breast cancer and advanced serous ovarian cancer [38,39]. Consistent with previous research, in the present study, AURKA showed a high expression in the DLBCL tissues and DLBCL cells, suggesting that AURKA may play a significant role in the DLBCL.

Cell viability could reflect the number of cells and is involved in cellular behaviors [40]. One of the fundamental approaches in the cancer treatment is to promote apoptosis of cancer cells [41]. According to a previous study, the apoptosis of DLBCL cells could be induced by the inhibition of Aurora kinase [19]. In the present study, the DLBCL cell viability was reduced, yet the cell apoptosis was increased by the silencing AURKA, mirroring that AURKA could act as a therapeutic target for DLBCL.

DLBCL is a non-Hodgkin lymphoma, and CHOP therapy is a regular method for non-Hodgkin lymphoma treatment [8,9]. For DLBCL, the CHOP has been reported to improve the prognosis of patients [10]. The AURKA inhibitor has presented preclinical synergy effects on the immunochemotherapy of DLBCL with rituximab and vincristine [20]. However, the effects of CHOP on AURKA have not been discussed, and there are few discussions about the effects of AURKA on CHOP treatment, and these were studied in the present study. According to the results of our experiments, CHOP reduced the viability of DLBCL cells and had no obvious regulative effects on the expression of AURKA in DLBCL cells, and CHOP could promote the apoptosis of DLBCL cells and silencing AURKA enhanced the effects of CHOP on promoting apoptosis of DLBCL cells, displaying that silencing AURKA could reinforce the therapeutic effects of CHOP on DLBCL.

The corresponding mechanism of the effects of AURKA on CHOP therapy remained obscure, which was also discussed in the present study. As a kind of multifunctional protein, the β-Catenin plays a pivotal role in modulating the homeostasis, and the regulation of β-Catenin activity has been regarded as a potential target of cancer treatment [42]. In DLBCL, the upregulation of β-Catenin could induce the progression of DLBCL [43]. In gastrointestinal cancers, the overexpression of AURKA could activate the β-Catenin signaling [15]. Furthermore, the interaction between β-Catenin and RAS-ERK pathway plays critical parts in the progress of various cancers, and by suppressing the β-Catenin pathway, the β-Catenin and RAS levels were degraded [44]. RAS has been viewed as a causal factor of cancers and drives the research of the effective RAS inhibitor in these years [45]. For instance, the inhibition of RAS pathway presents the anti-cancer activity to DLBCL [46]. In colorectal cancer, the RAS signaling has been reported to be activated by AURKA [18]. ERK1/2 controlled by the RAS modulates cell cycle, proliferation and development [25,47]. The activation of ERK1/2 contributes to the development of DLBCL [48]. Moreover, in breast cancer, the cancer cell proliferation is enhanced by AURKA via regulating ERK1/2 [19]. Nevertheless, there are few discussions about the factors related to AURKA in the CHOP therapy for DLBCL. According to our study, CHOP exerted no obvious effects on the expressions of RAS, p-β-Catenin, β-Catenin, p-ERK1/2 and ERK1/2. In comparison, silencing AURKA downregulated the expressions of RAS, β-Catenin and p-ERK1/2, but upregulated that of p-β-Catenin, and showed no prominent change in ERK1/2 level. Moreover, CHOP with silencing AURKA enhanced these effects of silencing AURKA on these factors above, indicating that CHOP combined with silencing AURKA had better effects on treating DLBCL via suppressing β-Catenin and RAS- ERK1/2 pathway.

However, there are still some limitations in our study. This research analyzed the effects and mechanism of AURKA on CHOP therapy for the treatment of DLBCL, but deeper mechanism of AURKA on CHOP therapy still needed to be researched and the corresponding treatment was necessary to be developed. Studies of deeper experiments *in vivo* about the corresponding mechanism are also necessary for further exploration.

## Conclusion

AURKA presented a high expression in DLBCL tissues and cells, and the silencing AURKA could reduce the viability and promote apoptosis of DLBCL cell. CHOP had no obvious effects on AURKA expression yet reduced viability and promoted apoptosis of DLBCL cells. These effects of CHOP on the viability and apoptosis of DLBCL cells could be enhanced by silencing AURKA via suppressing β-Catenin and RAS-ERK1/2 pathway. Collectively, silencing AURKA reinforced the therapeutic effects of CHOP on reducing viability and promoting apoptosis of DLBCL cell via suppressing β-Catenin and RAS-ERK1/2 pathway.

## Data Availability

The analyzed data sets generated during the study are available from the corresponding authors on reasonable requests.

## Highlights

1. **AURKA expression was upregulated in DLBCL tissues and cells.**
2. **SiAURKA suppressed the cell viability but promoted the apoptosis in DLBCL cells.**
3. **SiAURKA strengthened the effects of CHOP on enhancing the apoptosis of DLBCL cells.**
4. **siAURKA reinforced the effects of CHOP via β-Catenin and RAS-ERK1/2 pathway.**
